# The difference in the survival rate of patients with metastatic renal cell carcinoma in the intermediate-risk group of the Memorial Sloan Kettering Cancer Center criteria

**DOI:** 10.18632/oncotarget.25554

**Published:** 2018-06-12

**Authors:** Satoshi Tamada, Taro Iguchi, Sayaka Yasuda, Minoru Kato, Takeshi Yamasaki, Tatsuya Nakatani

**Affiliations:** ^1^ Department of Urology, Osaka City University Graduate School of Medicine, Abeno-ku, Osaka 545-8585, Japan

**Keywords:** molecular targeted therapy, renal cell carcinoma, Memorial Sloan Kettering Cancer Center criteria, metastasis, intermediate risk

## Abstract

**Objectives:**

To investigate the necessity of stratifying patients in the intermediate-risk group of the Memorial Sloan Kettering Cancer Center (MSKCC) criteria in a real-world population of patients with metastatic renal cell carcinoma.

**Patients and Methods:**

We retrospectively analyzed 234 consecutively treated patients who had received molecular targeted drugs. We examined the difference between progression-free survival and overall survival among patients in the intermediate-risk group of MSKCC criteria. We divided the intermediate group into two subgroups as follows: patients positive for only one risk factor (Int-1) and those positive for two risk factors (Int-2) including performance status, serum hemoglobin level, time from diagnosis to treatment, and corrected calcium and lactate dehydrogenase levels. Next, we evaluated the association between the number of metastatic organs, the presence of pancreatic metastasis, Int-1 or Int-2 grouping, and overall survival.

**Results:**

The median overall survival was 41.2 months. The median overall survival of the favorable-, intermediate-, and poor-risk groups of the MSKCC criteria were 91.0, 33.6, and 15.2 months, respectively. Patient characteristics were similar between the Int-1 and Int-2 groups. Increased positivity for risk factors of MSKCC classification between the two groups was for performance status and serum hemoglobin level. Progression-free survival and overall survival of the Int-1 group were significantly higher than those of the Int-2 group. In Cox proportional stepwise multivariate analysis, the Int-1 and Int-2 classification was an independent risk factor for overall survival.

**Conclusion:**

Patients in the intermediate-risk group had different prognoses depending on the number of positive risk factors.

## INTRODUCTION

The survival rate of patients with metastatic renal cell carcinoma has improved remarkably since the introduction of molecular targeted drugs [1–4]. We classified the prognoses of patients with metastatic renal cell carcinoma (mRCC) and used the classification as an index in selecting a treatment approach. The Memorial Sloan Kettering Cancer Center (MSKCC) risk classification is a survival risk classification advocated by Motzer et al. [5] before treatment using molecular targeted drugs became common. This classification is reportedly associated with prognosis even in the era of molecular targeted drugs [6] and is still widely used. However, the International Metastatic Renal Cell Carcinoma Database Consortium (IMDC) risk classification advocated by Heng et al. [7] was established in the era of molecular targeted drugs and is also used as widely as the MSKCC risk classification. There is also another risk classification targeted for Japanese patients, which was remodeled to be used more practically by incorporating factors attributable to metastatic organs [8].

However, patients in the intermediate group accounted for about half of all the patients. Moreover, the intermediate-risk group involves 1 or 2 risk factors; thus, there is a great variability among the patients. Consequently, it has not been decided whether it is appropriate to treat the intermediate-risk group as one group. Sella et al. based on six clinical trials data consisting in well-selected patients treated with sunitinib only, reported the heterogeneity of patients identified as having intermediate risk using the MSKCC risk criteria [9].

In this study, therefore, we performed a retrospective analysis of patients treated with tyrosine kinase inhibitors and mammalian target of rapamycin inhibitor in the intermediate-risk group within a real-world Japanese population to investigate the necessity of stratifying patients in the intermediate-risk group. Optimal classification for Japanese population may predict treatment effect or investigate a treatment approach in the future.

## RESULTS

Table 1 shows the characteristics of the study population. Patients in the intermediate risk class accounted for about 50% of all risk classes. The median overall survival (OS) was 41.2 months (Supplementary Figure 1). The median OS of the favorable-, intermediate-, and poor-risk groups based on the MSKCC criteria were 91.0, 33.6, and 15.2 months, respectively (Supplementary Figure 2). Patients in the favorable-risk group had a significantly prolonged survival than those in the other groups (hazard ratio (HR) 0.22, 95% confidence interval (CI) 0.11-0.44, p<0.001). Conversely, patients in the poor-risk group had a significantly shorter survival time compared to those in the other groups (HR 3.04, 95% CI 1.95-4.74, p<0.001) and compared to those in the intermediate-risk group alone (HR 2.15, 95% CI 1.37-3.39). The median OS using the first-line treatments- sunitinib, sorafenib, and temsirolimus were 69.6, 33.6, and 11.8 months, respectively.

**Table 1:** Patients characteristics and treatments (N=234)

Age (years), median		67	range: 35-84	
		Number of patients		
Sex	Male	180		
	Female	54		
MSKCC	Favorable	50	21.4	%
	Intermediate	126	53.8	%
	Poor	49	20.9	%
	unknown	9	3.8	%
Number of metastatic organs	Single	118	50.4	%
	Multiple	116	49.6	%
Sites of metastasis	Lung	153		
	Lymph node	59		
	Bone	67		
	Pancreas	14		
	Liver	20		
	Brain	13		
Prior nephrectomy	Yes	219	93.6	%
	No	15	6.4	%
Molecular targeted agents				
1st-line	Sunitinib	137		
	Sorafenib	75		
	Pazopanib	10		
	Temsirolimus	12		
2nd-line	Everolimus	32		
	Axitinib	57		
	Sunitinib	25		
	Temsirolimus	11		
	Pazopanib	2		
	Nivolumab	5		
	Sorafenib	5		
3rd-line	Everolimus	18		
	Sunitinib	14		
	Axitinib	16		
	Sorafenib	11		
	Temsirolimus	11		
	Nivolumab	9		
	Pazopanib	4		

**Figure 1:**
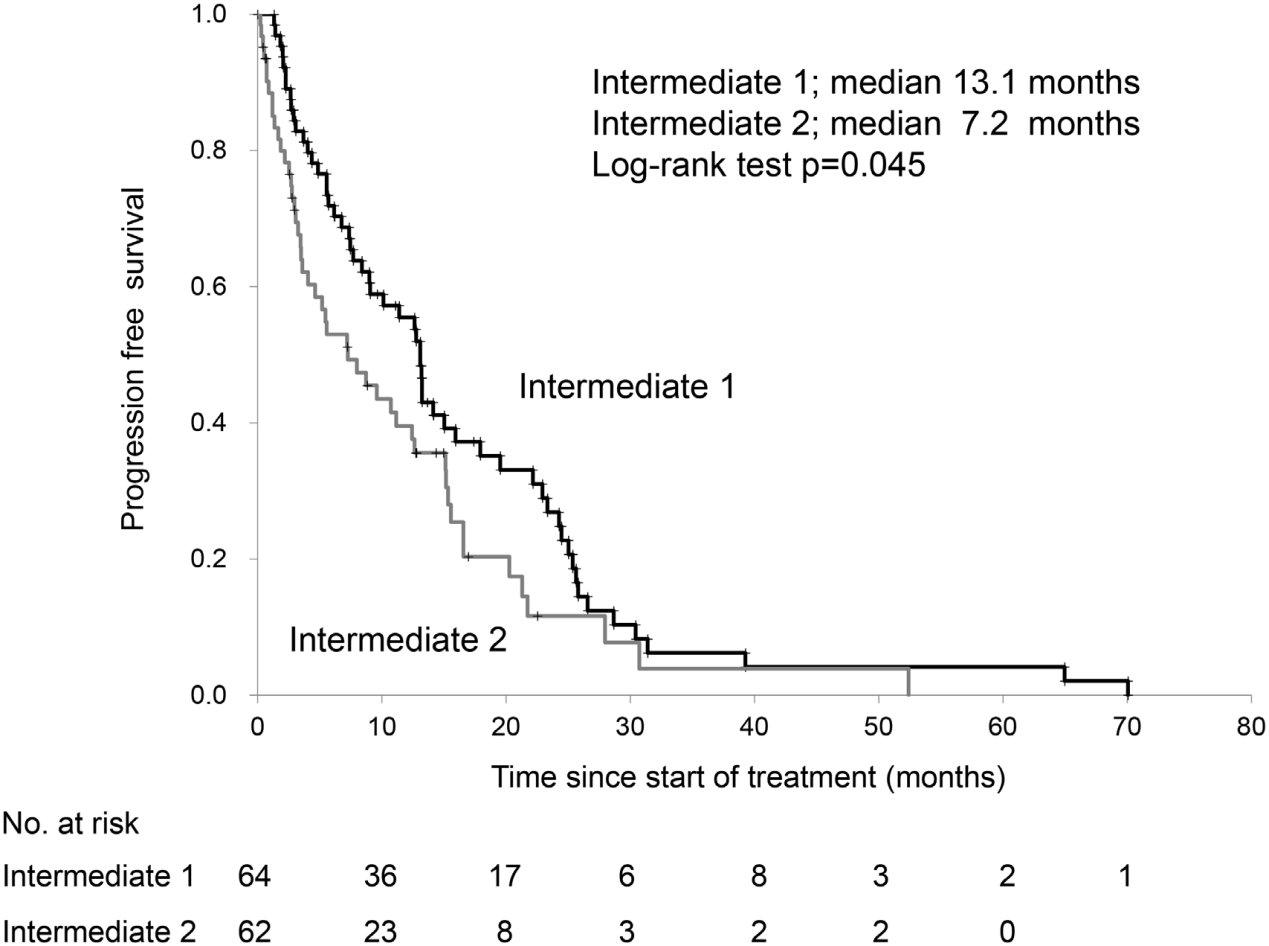
Progression-free survival of patients with 1 versus those with 2 risk factors in the Memorial Sloan Kettering Cancer Center risk classification

**Figure 2:**
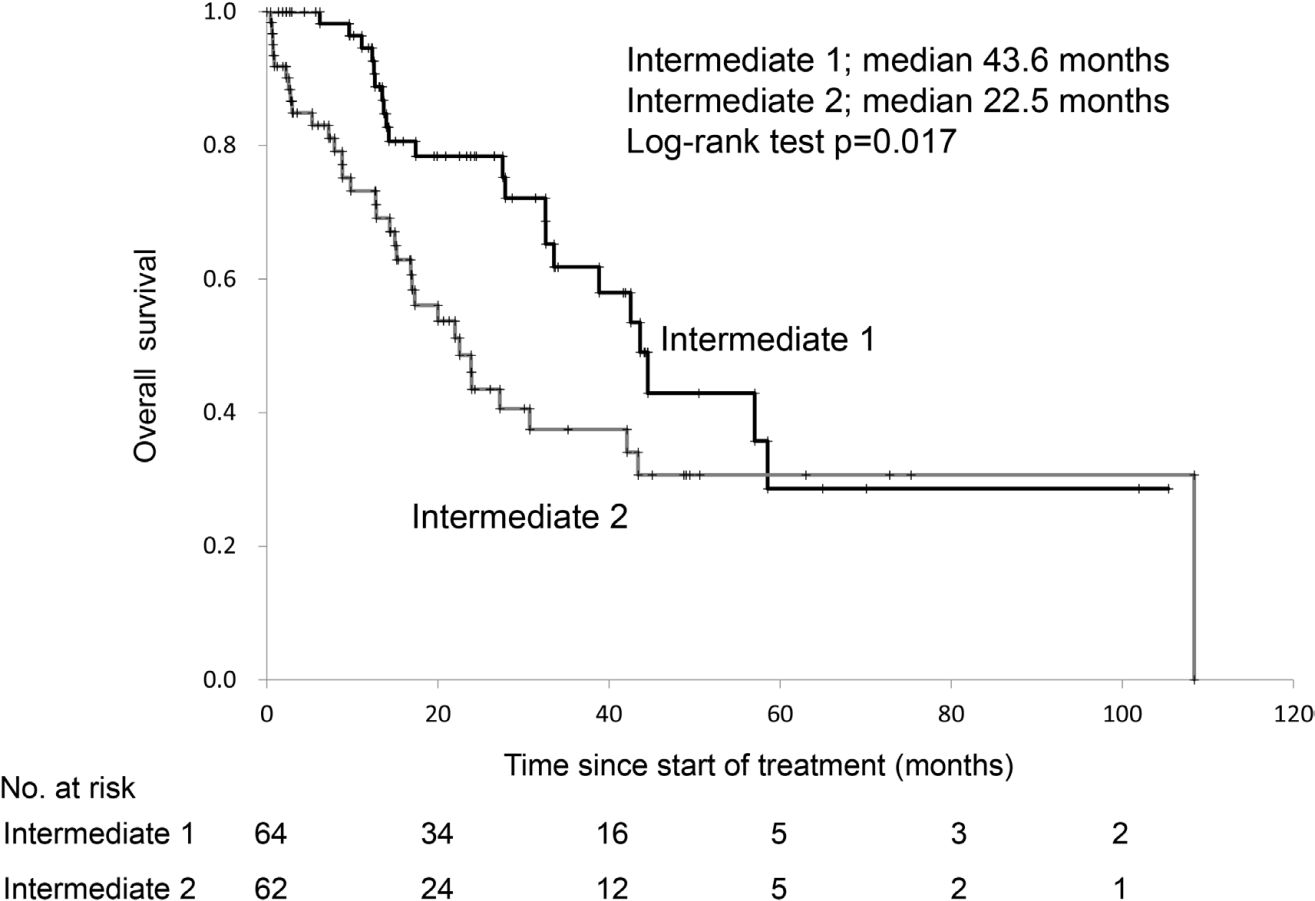
Overall survival of patients with 1 versus those with 2 risk factors in the Memorial Sloan Kettering Cancer Center risk classification

Table 2 shows the characteristics of patients in the intermediate-risk class. Patient characteristics were similar between the two subgroups. Increased positivity for risk factors of the MSKCC classification between Int-1 and Int-2 groups were for performance status and serum hemoglobin level. The median OS using the first-line treatments- sunitinib and sorafenib were 43.4 and 30.7 months, respectively, in the intermediate risk group (log-rank test, p=0.103). The progression-free survival (PFS) and OS of the Int-1 group were significantly higher than those of the Int-2 group (Figures 1, 2). Patients in the Int-1 group had a significantly shorter survival than those in the favorable group (HR 2.88, 95% CI 1.32-6.30, p=0.007). No significant difference in survival was found between the Int-2 and poor-risk groups (HR 1.42, CI 0.87-2.29, p=0.151). The median OS using the first-line drugs- sunitinib and sorafenib were 44.1 and 43.6 months, respectively, in the Int-1 group, (log-rank test, p=0.438) and were 27.2 and 15.2 months, respectively, in the Int-2 group, (log-rank test, p=0.099). We evaluated the risk factors of the MSKCC criteria to determine the risk factors that affect survival rate between the two groups, but no significant difference was observed in any of the risk factors (performance status (PS), 0 vs ≥1, HR 0.62, 95% CI 0.35-1.08; serum hemoglobin level, < lower limit of normal (LLN) vs ≥LLN, HR 1.41, 95% CI 0.82-2.43; time from diagnosis to treatment, <1 year vs ≥1 year, HR 1.47, 95% CI 0.85-2.52; corrected calcium level, <10 mg/dL vs ≥10 mg/dL, HR 0.40, 95% CI 0.15-1.08; lactate dehydrogenase (LDH) level, <1.5x upper limit of normal (ULN) vs 1.5xULN, HR 0.98, 95% CI 0.39-2.48).

**Table 2:** Patients characteristics and treatments in intermediate group (N=126)

		Intermediate 1		Intermediate 2	
		N=64		N=62	
Age (years), median		67	range: 40-80	69	range: 39-83
		Number of patients		Number of patients	
Sex	Male	53		46	
	Female	11		16	
Number of metastatic organs	Single	39		36	
	Multiple	25		26	
Sites of metastasis	Lung	46		45	
	Lymph node	12		16	
	Bone	18		15	
	Pancreas	4		2	
	Liver	3		4	
	Brain	2		2	
Positive factors in the Memorial Sloan Kettering Cancer Center criteria	Performance status	8		35	
	Serum hemoglobin level	16		36	
	Time from diagnosis to treatment	33		40	
	Corrected calcium level	4		6	
	Lactate dehydrogenase level	3		7	
1st-line treatment	Sunitinib	38		39	
	Sorafenib	23		16	
	Pazopanib	2		3	
	Temsirolimus	1		4	

Fifty-six out of the 126 patients in the intermediate group died. Cox proportional stepwise multivariate analysis showed that the Int-1 and Int-2 classification was an independent risk factor for OS (HR 1.91, 95% CI 1.11-3.30, p=0.019). (Table 3)

**Table 3:** Results of the Cox proportional stepwise multivariate analysis for the association between the variables and overall survival

Comparison			Overall survival (months), median			Unadjusted		Adjusted	
						HR (95% CI)	p value	HR (95% CI)	p value
Metastasis in a single organ	vs	Metastasis in multiple organs	38.8	vs	30.7	1.27 (0.74-2.18)	0.381	1.38 (0.80-2.38)	0.239
Int-1	vs	Int-2	43.6	vs	22.5	1.93 (1.12-3.33)	0.017	1.91 (1.11-3.30)	0.019
Presence of pancreatic metastasis	vs	Absence of pancreatic metastasis	32.6	vs	not reached	0.19 (0.02-1.40)	0.103	0.18 (0.02-1.38)	0.100

Abbreviations: HR, hazard ratio; CI, confidence interval.

## DISCUSSION

To the best of knowledge, we reported here for the first time an investigation of the necessity of stratifying patients in the intermediate-risk group of the MSKCC risk classification within a real-world Japanese population. We demonstrated that there was a significant difference in the survival period between Int-1 and Int-2 groups of the intermediate-risk group. This finding suggests that we need to subcategorize the intermediate-risk group to predict treatment effect or investigate a treatment approach in the future.

In addition to other studies, we previously reported that patients in the intermediate-risk group account for about 50% of patients with metastatic renal carcinoma and their survival rate falls between those of the favorable- and poor-risk groups [8, 10, 11]. However, a subcategorization of the intermediate-risk group has not been considered. In recent years, however, clinical trials have been conducted within the intermediate- and poor-risk groups alone or occasionally, clinical trials have been conducted to prove efficacy within these groups alone [12, 13]. Accordingly, a need has arisen to change the treatment approach to match the individual risk groups. However, since the intermediate-risk group involves 1 or 2 risk factors, there is a great variability among the patients and consequently, it has not been decided whether it is appropriate to treat the intermediate-risk group as one group. In our analysis, the OS of the Int-1 and Int-2 groups were 43.6 and 22.5 months, respectively, indicating that the prognosis of the latter group was significantly poorer (log-rank test, p=0.017). The most common positive risk factor in both groups was PS, followed by anemia. This finding suggests that the aggravation of PS and anemia may possibly have an influence on prognosis in both groups. Sella et al. [9] reported that they found a difference in OS and PFS between the Int-1 and Int-2 groups and in PFS and the objective response rate among patients with Eastern Cooperative Oncology Group PS ≥1. These analyses were compiled from the results of six clinical trials, and a conclusion was reached that segmenting the intermediate-risk group and stratification by PS are necessary. Our real-world data supports this conclusion. In addition, the mean survivals in the favorable- and poor-risk groups in the MSKCC study were 91.0 and 15.2 months, respectively. Since the difference in these groups was greater than that in the Int-1 group, prognosis prediction and treatment approach should be treated in a separate study group.

We showed in our previous study that the presence or absence of pancreatic metastasis and the number of metastatic organs, as well as the MSKCC classification, had an influence on prognosis [10]. However, Shinohara et al. [8] advocated a risk classification designed for Japanese patients. They defined the following four items as the stratification factors: hemoglobin; less than 1 year from diagnosis to treatment; LDH; and liver, bone, or multiple organs metastases. Consequently, they classified the favorable-risk group as 0-1 factor positive; the intermediate-risk group as 2 factors positive; and the poor-risk group as ≥3 factors positive. Compared to the MSKCC risk classification, this classification additionally incorporated the metastatic organs or the number of metastatic organs as stratification factors, but it was reported that its correlation with life prognosis was as favorable as that of the MSKCC risk classification. In a multivariate analysis to assess the number of metastatic organs, however, it was revealed that the number of metastatic organs was not an independent factor that could affect the survival period of patients in the Int-1 and Int-2 groups. This finding suggests that the number of positive risk factors is more important than the number of metastatic organs in the intermediate-risk group.

In the present study, OS was 41.2 months, which is longer than that in large-scale clinical trials [14, 15]. This may be influenced by the longer life expectancy of Japanese compared to other races. Furthermore, this may be due to the fact that Japanese have good treatment outcomes including other carcinomas [16]. However, Motzer et al. [17] reported that there were no differences in PFS or OS in Caucasian vs Asian patients. Conversely, there were significant differences in PFS and OS in Caucasians vs non-Caucasians, non-Asian patients. Several investigators also showed that the survival period after the introduction of molecular targeted drugs seemed prolonged compared to that before their introduction [1, 3, 4]. Of the first-line treatment drugs, the survival period of the patients who used sunitinib was the longest. The survival period of patients who used temsirolimus was remarkably short, probably due to its use in the poor-risk group.

There were some limitations to this study; we conducted a retrospective study and the treatment drugs were not unified. Most of the patients in this study were enrolled at a time before the introduction of immuno-oncology drugs in the clinical management of mRCC patients; thus, the survival period may prolong if immuno-oncology drugs become more widely used in the future [13, 18, 19]. In particular, treatment results of immuno-oncology drugs among patients in the poor-risk group are much awaited. Another limitation is that we reported here the necessity of stratifying patients in the intermediate-risk group using the MSKCC criteria. The IMDC criteria, which is another risk classification, is now often adapted to mRCC patients. However, some of our study participants had no available data regarding neutrophil counts, especially those who were diagnosed before 2013; hence, we did not investigate the IMDC risk. Although the MSKCC criteria was established in the cytokine therapy era, we have in addition to other authors, showed that it can be applied efficiently to Japanese patients, even in the era of molecular targeted drugs [8, 10].

In conclusion, we found that patients in the intermediate-risk group based on the MSKCC risk classification had different prognoses, depending on the number of positive risk factors. Our finding will be helpful in the selection of a future treatment approach.

## MATERIALS AND METHODS

We retrospectively analyzed 234 consecutively treated patients who had received molecular targeted drugs for mRCC. According to the therapeutic strategy in our institute, sorafenib was used as the first-line therapy and sunitinib or everolimus as the second-line therapy. However, since 2010, sunitinib has become the first-line therapy, and since 2012, axitinib has become the second-line therapy. We calculated OS and OS classified according to the MSKCC criteria. The OS period commenced from the time of treatment with the initial targeted therapy.

Next, we examined the difference between PFS and OS among patients in the intermediate-risk group of the MSKCC criteria. We divided the intermediate group into two subgroups as follows: patients who were positive for only one risk factor (Int-1) and those who were positive for two risk factors (Int-2) including performance status (PS), serum hemoglobin level, time from diagnosis to treatment, and corrected calcium and lactate dehydrogenase (LDH) levels. Subsequently, we investigated which factors had an influence on OS between the Int-1 and Int-2 groups.

OS was estimated using the Kaplan-Meier method and the differences were determined using the log-rank test. Cox proportional stepwise multivariate analysis was used to evaluate the association between the number of metastatic organs, Int-1 or 2 grouping, presence or absence of pancreatic metastasis, and OS. A p-value <0.05 was considered statistically significant. All statistical analyses were performed using Microsoft Excel^®^ (Microsoft, Redmond, Washington, USA). Permission to access the database for a review of the medical records of these patients was obtained from the local research ethics committee at Osaka City University (approval number 3441).

## SUPPLEMENTARY MATERIALS FIGURES



## Abbreviations

mRCC: metastatic renal cell carcinoma
MSKCC: Memorial Sloan Kettering Cancer Center
IMDC: International Metastatic Renal Cell Carcinoma Database Consortium
OS: overall survival
PFS: progression-free survival
PS: performance status
LDH: lactate dehydrogenase
HR: hazard ratio
CI: confidence interval
ULN: upper limit of normal
