# Supplementary material for: The difference in the survival rate of patients with metastatic renal cell carcinoma in the intermediate-risk group of the Memorial Sloan Kettering Cancer Center criteria
Source: Oncotarget. 2018 Jun 12;9(45):27752–9. doi: 10.18632/oncotarget.25554 (PMC6021254; doi:10.18632/oncotarget.25554)
Supplement: Supplementary file 1 [file oncotarget-09-27752-s001.pdf]

The difference in the survival rate of patients with metastatic renal cell carcinoma in the intermediate-risk group of the Memorial Sloan Kettering Cancer Center criteria

SUPPLEMENTARY MATERIALS

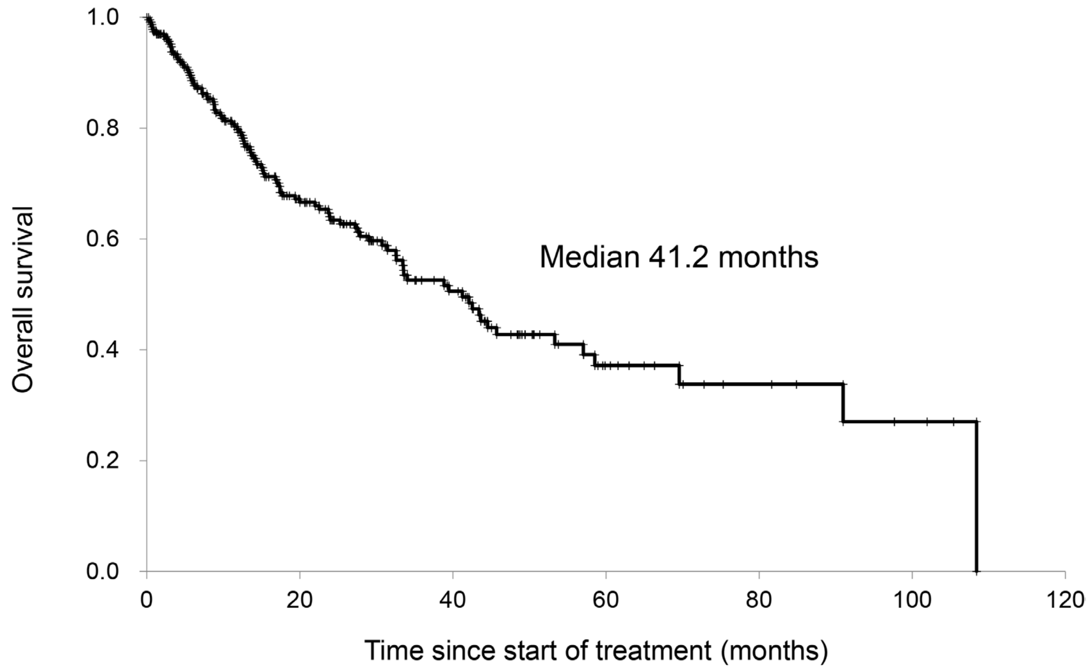

No. at risk 234 111 51 17 8 4

Supplementary Figure 1: Overall survival of the 234 patients with metastatic renal cell carcinoma.

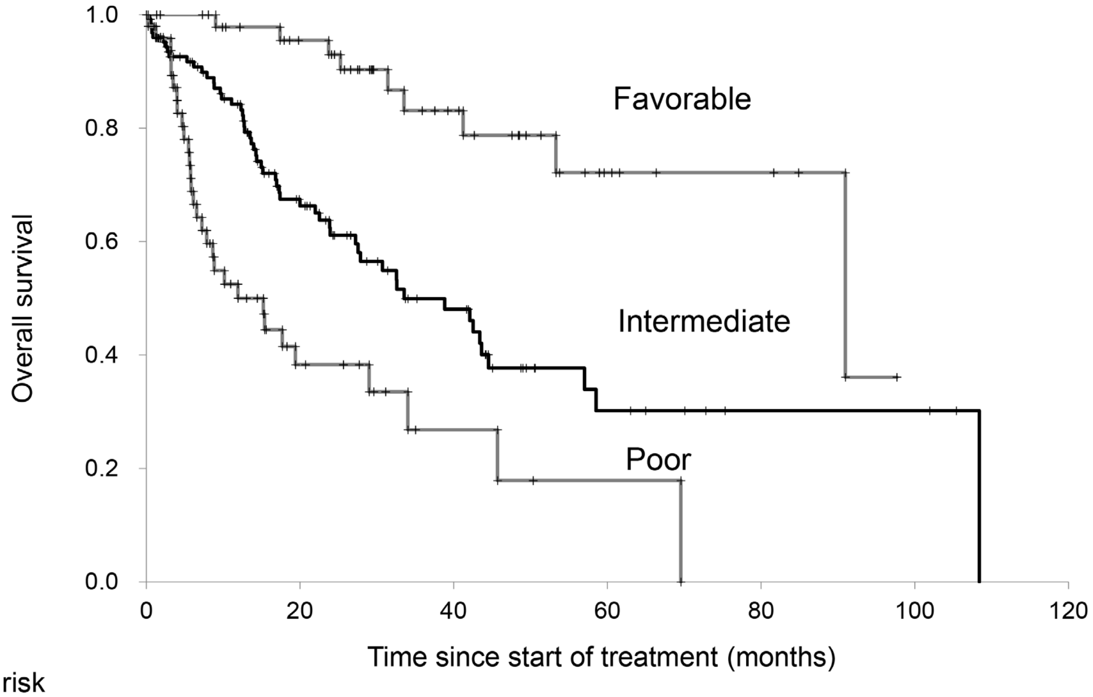

No. at risk						
Favorable	50	39	21	8	5	1
Intermediate	126	57	27	9	4	3
Poor	49	13	4	1	0	

Supplementary Figure 2: Overall survival stratified according to the Memorial Sloan Kettering Cancer Center risk classification into favorable-, intermediate-, and poor-risk groups.
